# Impact of Male Sex and Umbilical‐Pancreatic Anatomy on Surgical Difficulty in Minimally Invasive Distal Pancreatectomy: A Propensity‐Matched Analysis

**DOI:** 10.1111/ases.70284

**Published:** 2026-04-05

**Authors:** Masahiro Fukada, Noriki Mitsui, Takeshi Horaguchi, Itaru Yasufuku, Yuta Sato, Jesse Yu Tajima, Yoshihiro Tanaka, Nobuhisa Matsuhashi

**Affiliations:** ^1^ Department of Gastroenterological Surgery Gifu University Hospital Gifu Japan

**Keywords:** male, minimally invasive surgical procedures, propensity score

## Abstract

**Introduction:**

Minimally invasive distal pancreatectomy (MIDP), although demanding, offers clinical advantages. Male sex has been associated with increased operative difficulty; however, the anatomical basis for this disparity remains unclear. This study investigated sex‐related differences in MIDP outcomes using propensity score matching and evaluated physique‐related computed tomography (CT) indices potentially affecting MIDP complexity.

**Methods:**

Among 187 patients who underwent distal pancreatectomy between 2010 and 2024, 77 who received MIDP were analyzed. Propensity score matching incorporated body mass index (BMI), pancreatic cancer, buried splenic artery, transection line, spleen preservation, and surgical approach. Surgical outcomes were compared between sexes before and after matching. Preoperative CT was used to measure anatomical distances and calculate physique‐related indices, including the umbilical‐pancreatic ratio: pancreas‐umbilicus distance divided by xiphoid‐umbilicus distance.

**Results:**

After matching, 28 males and 28 females were included in the MIDP cohort. Males had longer operative times and a higher incidence of clinically relevant postoperative pancreatic fistula. CT analysis showed a greater pancreas‐to‐umbilicus distance and a higher umbilical‐pancreatic ratio in males. This ratio was correlated with BMI in males but not in females. Surgical difficulty, defined as operative time or intraoperative blood loss exceeding the 75th percentile, was independently associated with the umbilical‐pancreatic ratio, BMI, and pancreatic transection line on multivariable analysis.

**Conclusion:**

The increased surgical difficulty observed in male patients undergoing MIDP may be explained by the positional relationship between the umbilicus and the pancreas, particularly in patients with high BMI, rather than by sex alone. Cephalad camera port positioning may therefore be beneficial, especially in high‐BMI male patients.

**Abbreviations**
BMI: body mass index
CI: confidence interval
CR‐POPF: clinically relevant postoperative pancreatic fistula
CT: computed tomography
DP: distal pancreatectomy
JHPBS: Japanese Society of Hepato‐Biliary‐Pancreatic Surgery
JSES: Japan Society for Endoscopic Surgery
LDP: laparoscopic distal pancreatectomy
MIDP: minimally invasive distal pancreatectomy
MIS: minimally invasive surgery
ODP: open distal pancreatectomy
OR: odds ratio
PSM: propensity score matching

## 1 Introduction

Distal pancreatectomy (DP) is a key surgical approach for pancreatic body and tail disorders. Given that this procedure does not require reconstruction of the pancreatic or biliary tract, it is generally considered well‐suited for minimally invasive surgery (MIS) [1, 2, 3, 4, 5, 6]. Furthermore, compared with open surgery, minimally invasive DP (MIDP) offers reduced intraoperative blood loss, quicker recovery, lower postoperative complications and mortality, and a shorter hospital stay [7, 8]. Despite these surgical benefits, MIDP remains technically demanding. Several studies have identified perioperative factors influencing MIDP difficulty [8, 9, 10, 11, 12].

Specifically, factors such as male sex, body mass index (BMI), tumor location in the pancreatic body, pancreatic transection line, surgery for pancreatic cancer, parenchymal thickness at the pancreatic transection line, and a buried splenic artery have been independently associated with increased MIDP difficulty. While many of these factors are common in open distal pancreatectomy (ODP), some are more specific to or more impactful in MIDP. In our previous study [12], in ODP cases, high BMI, tumor location in the pancreatic body, and a buried splenic artery were significantly associated with prolonged operations. In contrast, male sex was a strong risk factor for prolonged surgery duration in MIDP.

Previous studies have identified male sex as a pure surgical risk factor for MIDP [8, 9, 10, 11], suggesting it may independently increase surgical difficulty, beyond confounding factors such as BMI and visceral fat. However, the mechanism behind this effect remains unclear. We hypothesized that the anatomical relationship between the umbilicus and pancreas influences surgical ergonomics and instrument reach in MIDP, potentially explaining the increased surgical difficulty observed in male patients.

Therefore, herein, we evaluated the effect of male sex on the surgical outcomes of MIDP by propensity score matching (PSM) analysis and identified indices related to physique by evaluating preoperative computed tomography (CT) scans that may negatively influence surgical outcomes.

## 2 Materials and Methods

### 2.1 Study Design, Aim, and Setting

This single‐center retrospective study evaluated sex differences in surgical outcomes in MIDP cases and sought to identify a physique‐related index for evaluating preoperative CT associated with technical difficulty. Surgical difficulty was assessed by operative time and intraoperative blood loss.

This retrospective study included patients who underwent DP for pancreatic diseases at the Department of Gastroenterological Surgery, Gifu University Hospital, between January 2010 and October 2024. All‐DP procedures were performed by expert surgeons certified by the Japanese Society of Hepato‐Biliary‐Pancreatic Surgery (JHPBS). MIDP procedures were conducted by qualified surgeons certified by the Japan Society for Endoscopic Surgery (JSES) endoscopic surgical skill qualification system. This study was conducted in accordance with the principles of the Declaration of Helsinki and approved by the Ethics Committee of Gifu University (approval number: 2024‐102).

### 2.2 Type of Surgery

Our institution performs laparoscopic distal pancreatectomy (LDP). Initially, this approach was limited to benign or low‐grade malignant tumors, in accordance with insurance coverage, with medications expanded to include malignant diseases in 2016. Since 2023, robotic‐assisted surgery (da Vinci Xi robotic system; Intuitive Surgical, Sunnyvale, CA, USA) has been adopted for all DP procedures, except for those requiring multi‐organ resection. In both approaches, the camera port was placed at the umbilicus.

### 2.3 Definition of Indices Related to Physique Evaluating Preoperative CT

Preoperative CT was used to measure anatomical distances between the following landmarks:

- Spina iliaca anterior superior.
- Xiphoid process to pubic symphysis.
- Xiphoid process to the umbilicus.
- Upper edge of the pancreas to the umbilicus.

**FIGURE 1:**
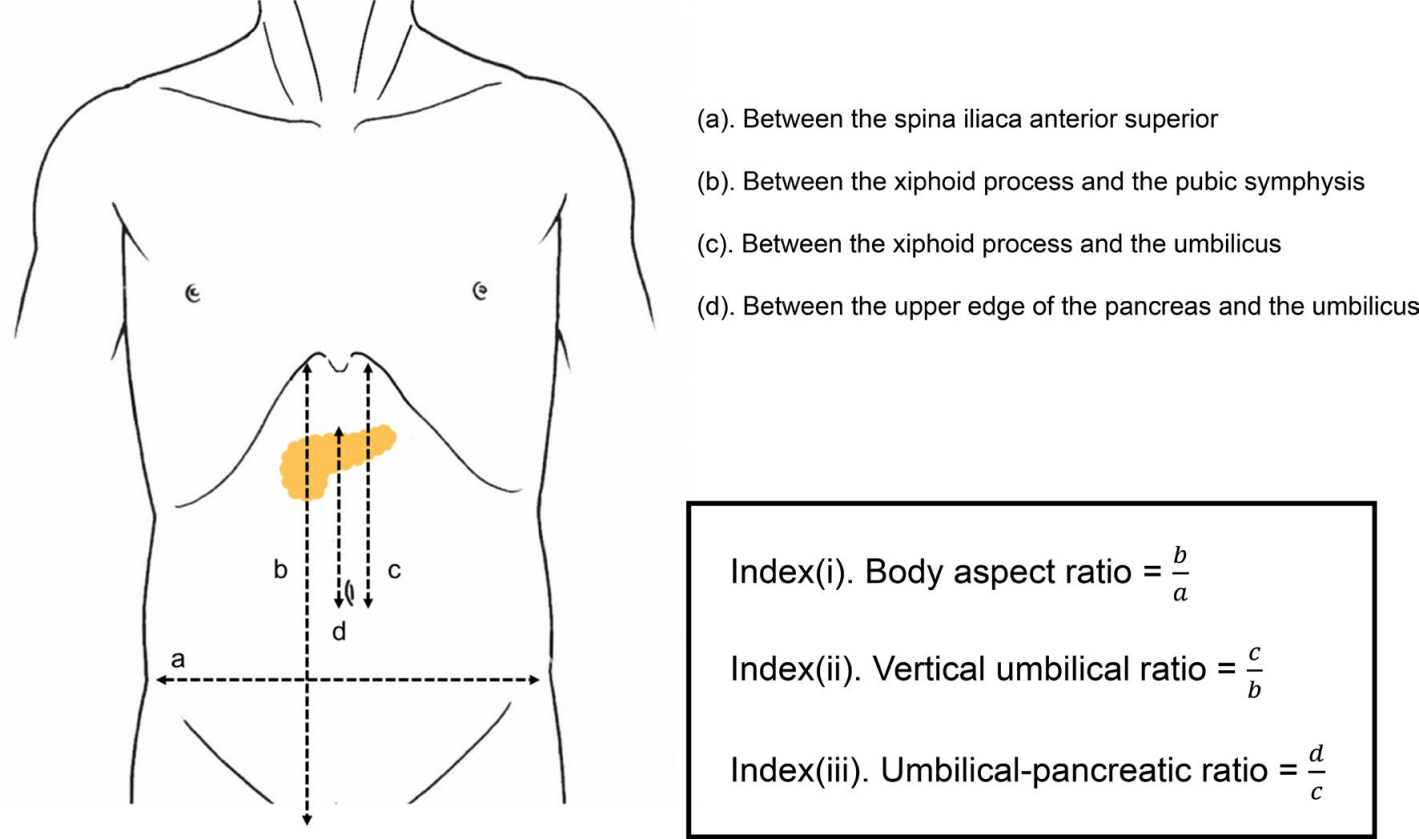
Definition of indices related to physique evaluating preoperative CT.

Next, physique‐related indices were calculated using the distances mentioned above (Figure 1).

Index (i). Body aspect ratio = (𝑏)/(𝑎).

Index (ii). Vertical umbilical ratio = (𝑐)/(𝑏).

Index (iii). Umbilical‐pancreatic ratio = (𝑑)/(𝑐).

## 3 Statistical Analysis

PSM at a 1:1 ratio was used to compare surgical outcomes between male and female patients. Propensity scores were calculated using logistic regression, and each male patient was matched to a female patient such that the match had the closest estimated propensity score within a caliper width of 0.20. Groups were matched on (1) BMI, (2) pancreatic cancer, (3) buried splenic artery, (4) pancreatic resection line (portal vein or left side of the aorta), (5) spleen preservation, and (6) type of surgery (laparoscopic or robotic surgery). These six covariates for PSM were selected based on clinical relevance and data availability. To validate the matching results, Fisher's exact test was used to compare categorical variables, and the Mann–Whitney *U* test was used to compare continuous variables between independent groups. Spearman's rank correlation coefficient was used to determine the correlation between two continuous variables. To evaluate risk factors for surgical difficulty in MIDP cases, surgically difficult cases were defined as those with operative time or blood loss exceeding the 75th percentile in this cohort. Variables with a *p* value < 0.05 in the univariate analysis were included in the multivariate logistic regression model.

Statistical significance was set at a two‐sided *p* value < 0.05. All statistical analyses were performed using JMP software (SAS Institute Inc., Cary, NC, USA).

## 4 Results

### 4.1 Patients

**FIGURE 2:**
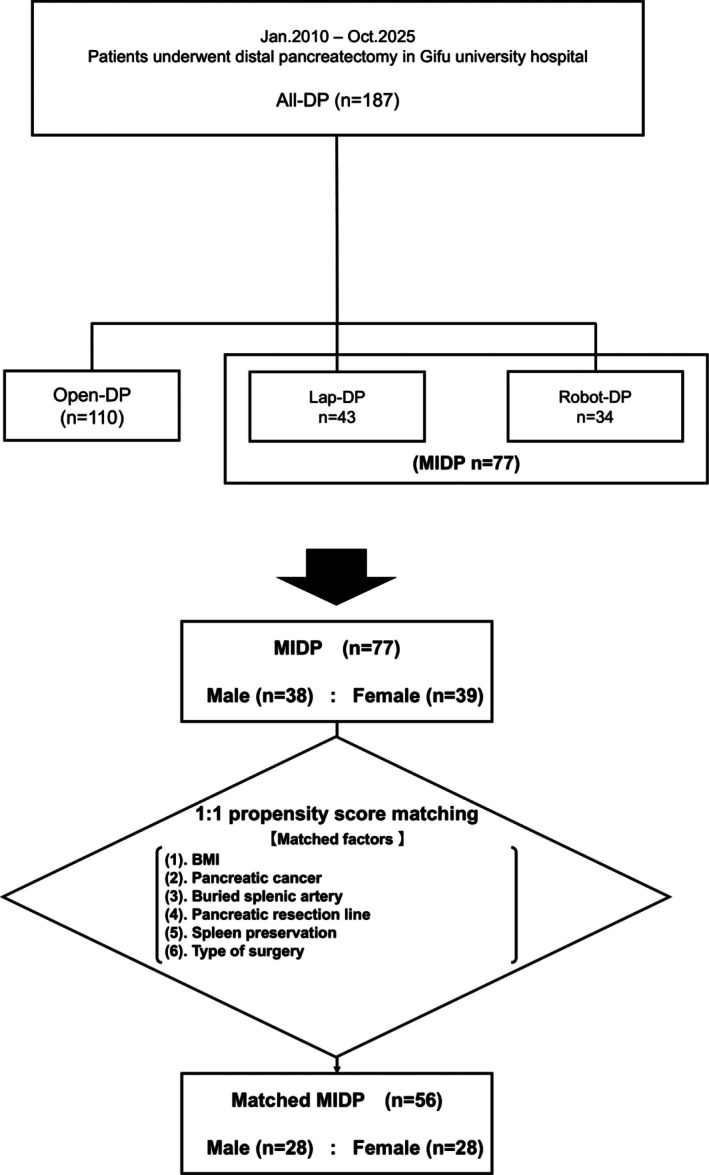
Study flow chart.

Overall, 187 patients who underwent DP for pancreatic disease were included in this study. Of these, 110 (58.8%) underwent ODP, and 77 (41.2%) underwent MIDP, including 43 laparoscopic and 34 robotic procedures (Table S1). To adjust for potential confounding variables between male and female cohorts, propensity score matching was performed for MIDP cases. Matching was performed between the cohorts in a 1:1 ratio. Following matching, the MIDP group comprised 28 males and 28 females (Figure 2).

### 4.2 Comparison of Perioperative Factors and Surgical Outcomes Between Males and Females in MIDP Cases Before and After PSM

Table 1 summarizes comparisons of perioperative factors, surgery, and surgical outcomes stratified by sex in MIDP cases. Before matching, males exhibited significantly higher BMI (*p* = 0.03) and experienced significantly longer operative times (*p* < 0.001), greater blood loss (*p* = 0.04), and extended hospital stays (*p* = 0.01). After PSM, baseline characteristics were balanced between sexes; males demonstrated significantly longer operative times (*p* < 0.001) and a higher incidence of clinically relevant postoperative pancreatic fistula (CR‐POPF) (*p* = 0.04) compared with females.

**TABLE 1:** Comparison of perioperative factors and surgical outcomes between male and female in MIDP cases before and after 1:1 propensity score matching.

MIDP cases	Before score matching (*n* = 77)			After score matching (*n* = 56)		
	Male	Female	*p*	Male	Female	*p*
	*n* = 38	*n* = 39		*n* = 28	*n* = 28	
Patient related factors						
Age (years)	68 [61–74]	66 [49–76]	0.23	68 [58–74]	68 [61–77]	0.82
BMI (kg/m^2^)	23.6 [21.1–25.3]	21.5 [19.0–23.6]	0.03*	22.5 [21.1–25.3]	22.4 [20.0–24.4]	0.68
Type of disease	Cancer: 18 (47.4%) Others: 20 (52.6%)	Cancer: 12 (30.8%) Others: 27 (69.2%)	0.14	Cancer: 10 (35.7%) Others: 18 (64.3%)	Cancer: 12 (42.9%) Others: 16 (57.1%)	0.58
Tumor location	Body: 15 (39.5%) Tail: 23 (60.5%)	Body: 14 (35.9%) Tail: 25 (64.1%)	0.75	Body: 11 (39.3%) Tail: 17 (60.7%)	Body: 9 (32.1%) Tail: 19 (67.9%)	0.58
Buried splenic artery	15 (39.5%)	16 (41.0%)	0.89	10 (35.7%)	13 (46.4%)	0.42
Parenchymal thickness at the transection line (mm)	13 [11–18]	13 [10–17]	0.87	14 [12–19]	13 [10–16]	0.24
Surgery related factors						
Pancreatic resection line	Portal vein: 22 (57.9%) Left side of aorta: 16 (42.1%)	Portal vein: 19 (48.7%) Left side of aorta: 20 (51.3%)	0.42	Portal vein: 13 (46.4%) Left side of aorta: 15 (53.6%)	Portal vein: 15 (53.6%) Left side of aorta: 13 (46.4%)	0.59
Spleen preservation	9 (23.7%)	11 (28.2%)	0.65	9 (32.1%)	8 (28.6%)	0.77
Robotic surgery	18 (44.4%)	16 (41.0%)	0.56	11 (39.3%)	10 (35.7%)	0.78
Surgical outcomes						
Operation time (min)	366 [305–452]	283 [264–319]	< 0.001***	338 [264–445]	283 [265–328]	0.04*
Blood loss (mL)	20 [4–101]	5 [0–55]	0.04*	20 [5–114]	10 [0–73]	0.19
CR‐POPF	6 (15.8%)	2 (5.3%)	0.13	4 (14.3%)	0 (0.0%)	0.04*
Hospital stay (days)	15 [13–20]	12 [11–14]	0.01*	15 [11–15]	13 [11–15]	0.12

*Note:* Data are expressed as median (interquartile range) or number of patients (percentage).

Abbreviations: BMI, body mass index; CR‐POPF, clinically relevant postoperative pancreatic fistula; DP, distal pancreatectomy.

*p < 0.05; ***p < 0.001.

### 4.3 Comparison of Indices Related to Physique Evaluating Preoperative CT Between Males and Females Before and After PSM

Table 2 summarizes distances between anatomical landmarks and physique‐related indices evaluated on preoperative CT stratified by sex. Males exhibited a significantly greater distance between the upper edge of the pancreas and the umbilicus (*p* < 0.01) and a significantly higher umbilical‐pancreatic ratio (*p* < 0.01) than females.

**TABLE 2:** Comparison of distances and indexes in preoperative CT between male and female in all DP cases.

	All cases (*n* = 187)		
	Male	Female	*p*
	*n* = 111	*n* = 76	
Distance between two anatomical landmarks			
(a). Left—right spina iliaca anterior superior (mm)	250 [239–263]	248 [236–259]	0.24
(b). Xiphoid process—pubic symphysis (mm)	353 [333–369]	354 [332–369]	0.45
(c). Xiphoid process—umbilicus (mm)	185 [171–204]	187 [169–201]	0.80
(d). Upper edge of the pancreas–umbilicus (mm)	129 [113–142]	121 [108–135]	< 0.01**
Indexes of anatomical landmarks			
(i). Body aspect ratio = (𝑏)/(𝑎)	1.40 [1.32–1.51]	1.40 [1.34–1.48]	0.95
(ii). Vertical umbilical ratio = (𝑐)/(𝑏)	0.53 [0.50–0.56]	0.53 [0.50–0.56]	0.62
(iii). Umbilical‐pancreatic ratio = (𝑑)/(𝑐)	0.69 [0.63–0.74]	0.67 [0.59–0.71]	< 0.01**

*Note:* Data are expressed as median (interquartile range) or number of patients (percentage).

**; *p* < 0.01.

### 4.4 Univariate and Multivariate Analyses of Risk Factors for Surgical Difficulty in MIDP Cases

Table 3 presents the univariate and multivariate analyses of risk factors for surgical difficulty in MIDP. According to the definition, 19 cases (24.7%) were classified as surgically difficult cases in this study. In the univariate analysis, surgical difficulty in MIDP cases was significantly associated with sex (*p* < 0.01), BMI (*p* = 0.01), the pancreatic resection line (*p* < 0.01), and the umbilical‐pancreatic ratio (*p* < 0.001). Multivariate logistic regression analysis revealed that BMI (> 24; OR 5.02; 95% CI: 1.31–22.62; *p* = 0.02), the pancreatic resection line (above the portal vein; OR 9.87; 95% CI: 2.40–54.23; *p* < 0.01), and the umbilical‐pancreatic ratio (> 0.62; OR 6.68; 95% CI: 1.27–53.75; *p* = 0.02) were independently associated with surgical difficulty in MIDP cases.

**TABLE 3:** Univariate and multivariate analysis of risk factors for surgical difficulty in MIDP cases.

	*n*	Univariate			Multivariate		
		OR	95% CI	*p*	OR	95% CI	*p*
Sex							
Male	38	5.71	1.81–22.01	< 0.01**	2.98	0.72–14.06	0.13
Female	39	1			1		
BMI (kg/m^2^)							
24	26	3.94	1.35–12.06	0.01*	5.02	1.31–22.62	0.02*
24	51	1			1		
Pancreatic cancer							
Yes	30	2.11	0.74–6.16	0.16			
No	47	1					
Buried splenic artery	31	1.96	0.69–5.69	0.21			
Yes	46	1					
No							
Pancreatic resection line							
Portal vein	41	4.61	1.47–17.74	< 0.01**	9.87	2.40–54.23	< 0.01**
Left side of aorta	36	1			1		
Spleen preservation							
Yes	20	0.7	0.18–2.28	0.57			
No	57	1					
Type of surgery							
Laparoscopic	43	0.64	0.22–1.80	0.39			
Robotic	34	1					
Umbilical‐pancreatic ratio							
> 0.62	51	5.75	1.46–38.47	0.01*	6.68	1.27–53.75	0.02*
< 0.62	25	1			1		

Abbreviations: BMI, body mass index; DP, distal pancreatectomy.

*p < 0.05; **p < 0.01.

### 4.5 Comparison of the Correlation Between BMI and the Umbilical‐Pancreatic Ratio Across Sexes

**FIGURE 3:**
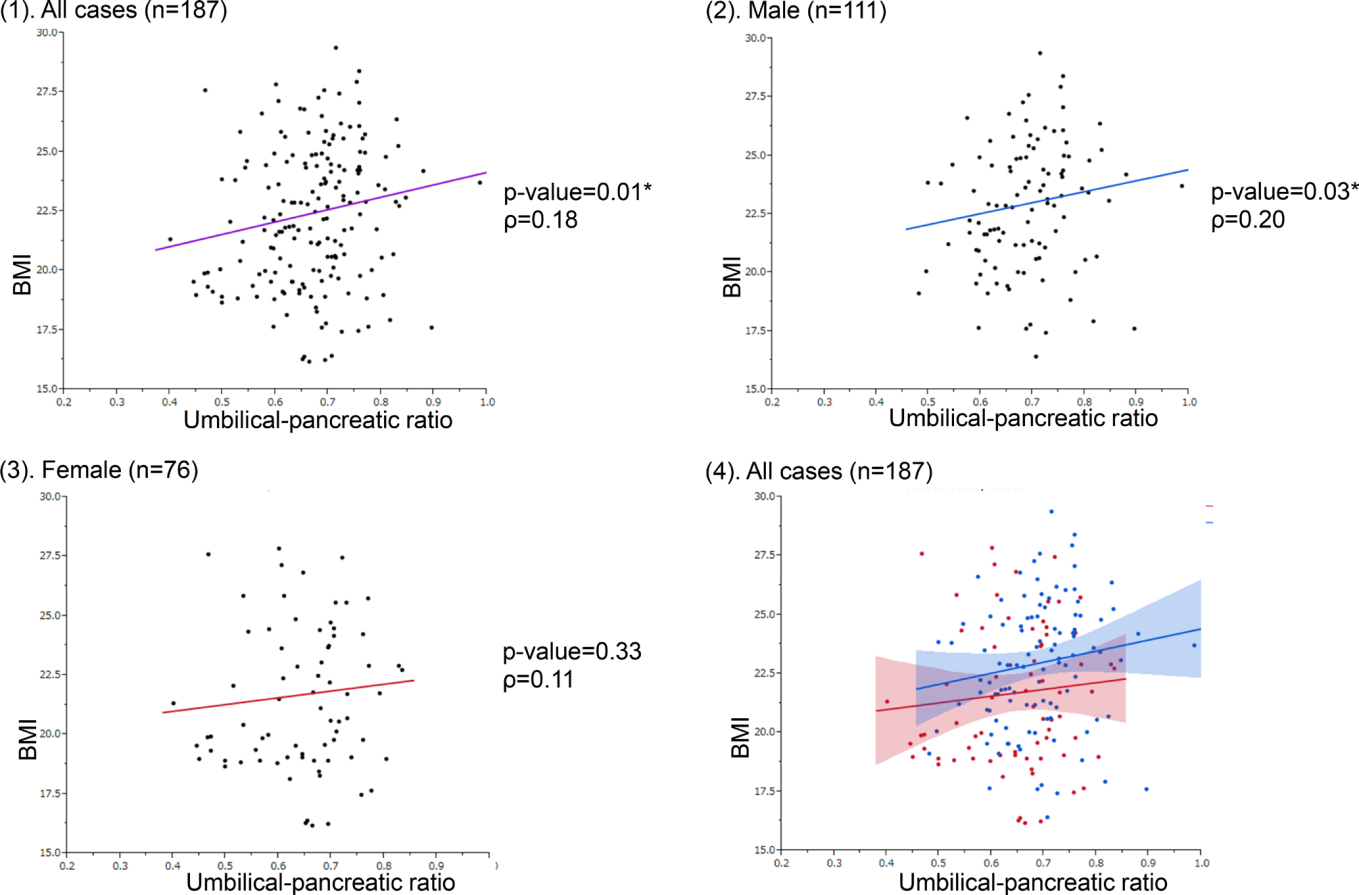
Comparison of the correlation between BMI and the umbilical‐pancreatic ratio across sexes.

Figure 3 shows the correlation between BMI and the umbilical‐pancreatic ratio across the sexes. In males, BMI and the umbilical‐pancreatic ratio showed a significant positive correlation (*p* = 0.03, *p* = 0.20). In contrast, no significant correlation was observed in females (*p* = 0.33).

## 5 Discussion

MIS has become increasingly common in gastroenterological surgery, including technically demanding hepatobiliary‐pancreatic surgery. Among these, DP is particularly amenable to MIS because the operative steps are comparatively well standardized. The first laparoscopic distal procedure was reported by Gagner et al. [13] in 1996, followed by the introduction of the robotic approach by Giulianotti et al. [14] in 2003. Since then, MIDP, including both laparoscopic and robotic techniques, has gained widespread acceptance. However, MIDP requires surgical techniques that are distinct from ODP, and understanding the inherent risk factors influencing surgical difficulty is essential to fully realize its benefits.

In our previous study [12], factors contributing to prolonged operative time differed between ODP and MIDP, with male sex emerging as a strong risk factor for MIDP. Previous studies have reported findings consistent with our results, indicating that male sex has a significant impact on surgical difficulty, regardless of whether MIDP is performed laparoscopically or robotic‐assisted [10, 11]. In this study, PSM was employed to assess whether sex is a genuine risk factor for surgical difficulty. Interestingly, when limited to MIDP cases, males had significantly longer operative times and a higher incidence of CR‐POPF than females. The generally accepted explanation for this result is that male patients have a different body fat composition with increased intra‐abdominal visceral fat compared to females, which reduces operative and complicates instrument manipulation [15, 16]. Visceral fat area (VFA) provides a more accurate assessment of intra‐abdominal fat volume and body shape than BMI. Elevated VFA levels have also been linked to a higher risk of postoperative complications following abdominal surgical procedures [17, 18].

**FIGURE 4:**
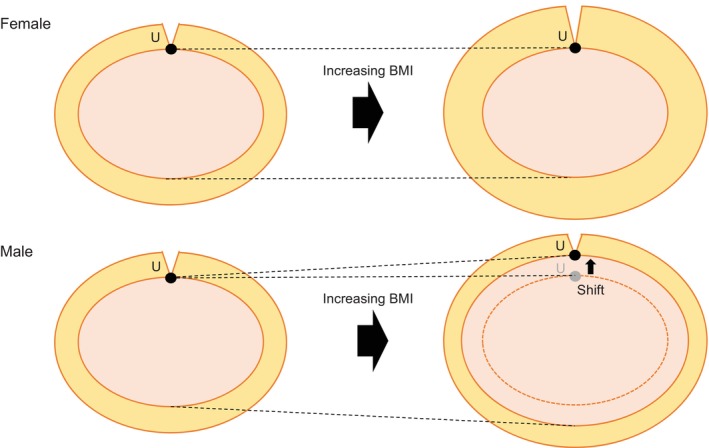
Differential models in umbilical position shift associated with increasing BMI in males and females.

In this study, we examined factors beyond VFA by evaluating anatomical distances and physique‐related indices on preoperative CT. We demonstrated for the first time that males had a significantly longer distance between the upper edge of the pancreas and the umbilicus and a higher umbilical‐pancreatic ratio than females. Interestingly, no significant sex differences were observed in several other distances or indices. In addition, multivariate analysis of surgical difficulty demonstrated that sex was not a significant factor, whereas the umbilical‐pancreatic ratio, along with high BMI, remained statistically significant. This result may explain why the male sex has a negative impact on surgical difficulty in MIDP. One possible mechanism is that in MIS, an increased distance from the umbilicus to the pancreas adversely affects an endoscopic surgical view during peripancreatic maneuvers, trocar reach, and impairs instrument angulation. Therefore, we consider that the umbilical‐pancreatic ratio has two potential advantages over VFA. First, it is a simple and easily obtainable index compared with VFA. Second, unlike VFA, the umbilical‐pancreatic ratio may be directly applicable to surgical planning, as the measurement can potentially be used to optimize port placement. Furthermore, we demonstrated for the first time that the umbilical‐pancreatic ratio exhibits a significant positive correlation with BMI exclusively in males. Regarding this result, we hypothesized that as BMI increases, females experience an increase in subcutaneous fat, whereas males experience an increase in visceral fat; consequently, the male umbilicus shifts ventrally (Figure 4). Thus, male sex and high BMI may synergistically increase surgical difficulty through their combined influence on the anatomical relationship between the umbilicus and pancreas. In robotic surgery, the only available endoscope has a 30‐degree oblique view, which may exacerbate the issue. Our findings suggest that this limitation may be mitigated by positioning the camera port towards the head, specifically at approximately 20 mm in male patients with elevated BMI. This 20‐mm adjustment was based on our finding that the absolute umbilicus‐pancreas distance in high‐BMI male patients was approximately 20 mm longer than the median value of the study cohort.

This study had some limitations. It was a single‐center retrospective study with a small sample size, which may have introduced selection bias and affected statistical power. Second, the potential negative impact of the umbilical‐pancreatic ratio on surgical difficulty remains a hypothesis; future studies are required to evaluate the possible association between changing the camera port sites and surgical outcomes. These limitations should be considered when interpreting the results. Prospective multicenter studies with a larger number of patients are required to validate our findings.

In conclusion, male sex represents a genuine risk factor for surgical difficulty in MIDP, with an increased umbilical‐pancreatic ratio potentially contributing to this result. This index has the significant advantage of being easier to calculate compared to VFA. Additionally, in male patients and those with a high BMI, shifting the camera port position based on this index cephalad may also reduce surgical difficulty.

## Author Contributions

Masahiro Fukada conceived and designed the study, interpreted the results, and drafted the manuscript. Nobuhisa Matsuhashi critically revised the manuscript for intellectual content. Masahiro Fukada, Noriki Mitsui, Takeshi Horaguchi, Itaru Yasufuku, Yuta Sato, Jesse Yu Tajima, Yoshihiro Tanaka, and Nobuhisa Matsuhashi acquired the data, provided critical comments, and approved the final manuscript.

## Funding

The authors have nothing to report.

## Ethics Statement

This study was conducted in accordance with the principles of the Declaration of Helsinki and approved by the Ethics Committee of Gifu University (approval number: 2024‐102). Given the retrospective nature of this study and the exclusion of potentially identifiable patient data, the requirement for informed consent was waived by the Ethics Committee of Gifu University.

## Consent

The authors have nothing to report.

## Conflicts of Interest

The authors declare no conflicts of interest.

## Supporting information


**Table S1:** Perioperative factors and surgical outcomes in all DP cases and MIDP cases in this study.

## Data Availability

The datasets generated in this study are available from the corresponding author upon reasonable request.
