# Supplementary material for: Impact of Male Sex and Umbilical‐Pancreatic Anatomy on Surgical Difficulty in Minimally Invasive Distal Pancreatectomy: A Propensity‐Matched Analysis
Source: Asian J Endosc Surg. 2026 Apr 5;19(1):e70284. doi: 10.1111/ases.70284 (PMC13050627; doi:10.1111/ases.70284)
Supplement: Supplementary file 1 — Table S1: Perioperative factors and surgical outcomes in all DP cases and MIDP cases in this study. [file ASES-19-e70284-s001.docx]

**Supplemental Table 1. Perioperative factors and surgical outcomes in all DP cases and MIDP cases in this study**

|  | All-DP | MIDP |
| --- | --- | --- |
|  | n=187 | n=77 |
| Patient related factor | | |
| Gender | Male: 111 (59.4%)  Female: 76 (40.6%) | Male: 38 (49.4%)  Female: 39 (50.7%) |
| Age (years) | 68 [60-75] | 67 [54-74] |
| BMI (kg/m^2^) | 22.3 [19.9-24.24.6] | 22.2 [19.5-24.8] |
| ASA | 1-2: 177 (94.7%)  3:10 (5.3%) | 1-2:75 (97.4%)  3:2 (2.6%) |
| Type of disease | Cancer: 95 (50.8%)  Others: 92 (49.2%) | Cancer: 30 (39.0%)  Others: 47 (61.0%) |
| Pathology | PDAC: 87 (46.5%)  Other cancer: 8 (4.3%)  MCN: 6 (3.2%)  SPN: 10 (5.3%)  SCN: 3 (1.6%)  NET: 29 (15.5%)  IPMN: 26 (13.9%)  Others: 18 (9.6%) | PDAC: 27 (35.1%)  Other cancer: 3 (3.9%)  MCN: 4 (5.2%)  SPN: 6 (7.8%)  SCN: 1 (1.3%)  NET: 24 (31.2%)  IPMN: 8 (10.4%)  Others: 4 (5.2%) |
| Tumor location | Body: 104 (55.6%%)  Tail: 83 (44.4%%) | Body: 29 (37.7%)  Tail: 48 (62.3%) |
| Tumor size (mm) | 24 [16-31] | 20 [12-30] |
| Buried splenic artery | 86 (46.0%) | 31 (40.3%) |
| Parenchymal thickness at the transection line (mm) | 13 [10-16] | 13 [11-18] |
| Surgery related factor | | |
| Pancreatic resection line | Portal vein: 121 (64.7%)  Left side of aorta: 66 (35.2%) | Portal vein: 41 (53.2%)  Left side of aorta: 36 (46.7%) |
| Spleen preservation | 26 (13.9%) | 20 (26.0%) |
| Type of surgery | Open: 110 (58.8%)  Minimally invasive surgery: 77 (41.2%) | Laparoscopic: 43 (55.8%)  Robot assisted: 34 (44.2%) |
| Surgical outcomes | | |
| Operation time (min) | 291 [245-367] | 315 [270-393] |
| Blood loss (ml) | 180 [20-544] | 10 [0-64] |
| Blood transfusion | 21 (11.2%) | 3 (3.9%) |
| CR-POPF | 39 (20.9%) | 8 (10.4%) |
| Hospital stay (days) | 14 [12-21] | 14 [11-17] |

Data are expressed as median (interquartile range) or number of patients (percentage).

DP: distal pancreatectomy

BMI: body mass index

ASA: American society of anesthesiologists

CR-POPF: clinically relevant postoperative pancreatic fistula

*: p< 0.05 **: p< 0.01 ***: p< 0.001
